# Ouabain Enhances ADPKD Cell Apoptosis via the Intrinsic Pathway

**DOI:** 10.3389/fphys.2016.00107

**Published:** 2016-03-24

**Authors:** Jessica Venugopal, Gustavo Blanco

**Affiliations:** Department of Molecular and Integrative Physiology and The Kidney Institute, University of Kansas Medical CenterKansas City, KS, USA

**Keywords:** Na, K-ATPase signaling, polycystic kidney disease, programmed cell death, BCL-2 proteins, cytochrome c, caspase

## Abstract

Progression of autosomal dominant polycystic kidney disease (ADPKD) is highly influenced by factors circulating in blood. We have shown that the hormone ouabain enhances several characteristics of the ADPKD cystic phenotype, including the rate of cell proliferation, fluid secretion and the capacity of the cells to form cysts. In this work, we found that physiological levels of ouabain (3 nM) also promote programmed cell death of renal epithelial cells obtained from kidney cysts of patients with ADPKD (ADPKD cells). This was determined by Alexa Fluor 488 labeled-Annexin-V staining and TUNEL assay, both biochemical markers of apoptosis. Ouabain-induced apoptosis also takes place when ADPKD cell growth is blocked; suggesting that the effect is not secondary to the stimulatory actions of ouabain on cell proliferation. Ouabain alters the expression of BCL family of proteins, reducing BCL-2 and increasing BAX expression levels, anti- and pro-apoptotic mediators respectively. In addition, ouabain caused the release of cytochrome c from mitochondria. Moreover, ouabain activates caspase-3, a key “executioner” caspase in the cell apoptotic pathway, but did not affect caspase-8. This suggests that ouabain triggers ADPKD cell apoptosis by stimulating the intrinsic, but not the extrinsic pathway of programmed cell death. The apoptotic effects of ouabain are specific for ADPKD cells and do not occur in normal human kidney cells (NHK cells). Taken together with our previous observations, these results show that ouabain causes an imbalance in cell growth/death, to favor growth of the cystic cells. This event, characteristic of ADPKD, further suggests the importance of ouabain as a circulating factor that promotes ADPKD progression.

## Introduction

Autosomal dominant polycystic kidney disease (ADPKD) is the most common genetic disorder of the kidney, characterized by the formation and progressive enlargement of numerous fluid filled cysts, which severely distort renal morphology and function, reviewed in Grantham (2008) and Paul and Vanden Heuvel (2014). ADPKD cysts are formed *in utero* and continue progressing after birth at a relatively slow, but relentless rate throughout the life of the affected individual (Grantham et al., 2010). Patients with ADPKD eventually develop renal insufficiency and end-stage renal disease (ESRD), requiring dialysis or kidney replacement therapy (Alam and Perrone, 2010; Grantham et al., 2011; Kanaan et al., 2014).

ADPKD is caused by mutations in the genes that encode for polycystin-1 and polycystin-2 (*Pkd1* and *Pkd2* respectively); however, progression of the disease is highly influenced by factors circulating in the bloodstream (Pei, 2011; Fedeles et al., 2014; Ong and Harris, 2015). We have shown that the hormone ouabain, in concentrations similar to those present in plasma, stimulate the proliferation of renal epithelial cells obtained from kidney cysts of patients with ADPKD (ADPKD cells), the growth of microcysts generated by ADPKD cells, and cyst-like tubule dilations in embryonic kidneys from a mouse model of ADPKD (Nguyen et al., 2007; Jansson et al., 2012). In contrast, ouabain does not significantly influence cell proliferation and cyst formation in normal kidney cells (NHK cells) and metanephric organs from wild type mice (Blanco and Wallace, 2013).

The slow progression of ADPKD is difficult to explain in a condition that is primarily characterized by continuous cell proliferation. Cell growth is maintained by a balance between cell proliferation and apoptosis, a process of programmed cell death (Green and Llambi, 2015; Savitskaya and Onishchenko, 2015). Interestingly, an imbalance between increased rates of cell apoptosis have been reported in kidneys from animal models of ADPKD and in humans carrying the disease, a phenomenon that may contribute to the uncontrolled, but slow progression of the disease (Lanoix et al., 1996; Zhou and Kukes, 1998; Murcia et al., 1999; Torres, 1999; Edelstein, 2005; Ibrahim, 2007; Goilav et al., 2008; Ibraghimov-Beskrovnaya and Bukanov, 2008).

Apoptosis is an essential process during normal tissue development and aging and is also found in several pathological situations (Elmore, 2007; Tezil and Basaga, 2014; Arya and White, 2015; Labi and Erlacher, 2015). Apoptosis involves an intricate cascade of molecular events, with the B-cell lymphoma 2 (BCL-2) protein family and a series of cysteine proteases, the caspases, being essential mediators of the process. The BCL-2 family include several members that are pro-survival and pro-apoptotic factors, such as BCL-2 and BAX respectively. The proteolytic caspases include the “initiator” caspases-8, -9, and -10, and the “executioner” caspases 3 and 7 (Elmore, 2007; Green and Llambi, 2015; Zheng et al., 2015). Two main caspase-mediated pathways control programmed cell death. The extrinsic pathway, a ligand triggered and transmembrane receptor mediated cascade (Ashkenazi, 2015), and the intrinsic pathway, which comprises mitochondrial changes and the release of cytochrome c from the mitochondrial intermembrane space to the cell cytosol (Brenner and Mak, 2009). Both intrinsic and extrinsic pathways converge to stimulate the activity of caspases-3 and -7, which are responsible for the events that are characteristic of apoptosis, including DNA fragmentation, protein cross-linking and degradation, and cell disintegration into apoptotic bodies (Salvesen and Riedl, 2008).

While apoptosis has been described as a feature of ADPKD, the factors and mechanisms that influence programmed cell death in ADPKD cells are poorly understood. Ouabain has been shown to influence programmed cell death in a cell type specific manner. For instance, ouabain has pro-apoptotic effects in normal neuronal cells, neuro- and glioblastoma cells, hepatic cells, blood peripheral lymphocytes, lymphoma cells, and prostate cancer cells (Olej et al., 1998; Xiao et al., 2002; Huang et al., 2004; Esteves et al., 2005; Kulikov et al., 2007; Panayiotidis et al., 2010; Xu et al., 2010; Fu et al., 2013; Yan et al., 2015). In contrast, ouabain protects endothelial cells, cerebellar granule cells, renal proximal tubule, and COS-7 cells against apoptosis (Isaev et al., 2000; Orlov et al., 2004; Trevisi et al., 2004, 2006; Li et al., 2010; Dvela et al., 2012; Burlaka et al., 2013); and has a dual pro- and anti-apoptotic effect in smooth muscle, umbilical vein endothelial cells, and fibroblast (Chueh et al., 2001; Winnicka et al., 2010; Ren et al., 2014). At present, the role of ouabain in ADPKD apoptosis is unknown. In this work, we show that ouabain enhances apoptosis in ADPKD, but not in NHK cells, by activating the intrinsic pathways of programmed cell death.

## Materials and methods

### Cell culture

Primary cell cultures of ADPKD cells, derived from surface cysts of ADPKD kidneys and NHK cells were generated by the PKD Biomaterial Core at University of Kansas Medical Center (KUMC). A protocol for the use of discarded human kidney tissues was approved by the Institutional Review Board at KUMC. Primary cultures were prepared as described (Wallace et al., 1996). Cells were seeded and grown in DMEM/F12 supplemented with 5% heat-inactivated fetal bovine serum (FBS), 100 IU/ml penicillin G and 0.1 mg/ml streptomycin, 5 μg/mL insulin, 5 μg/mL transferrin, and 5 ng/mL sodium selenite (ITS). Twenty-four hours before, cells were subjected to various experimental manipulations, serum was reduced to 0.002% and ITS removed. As previously shown, these cells are epithelial in nature and they stain positive for specific lectin markers for the collecting duct and distal nephron, indicating that they are derived from the distal nephron (Yamaguchi et al., 2003).

### Annexin-V and propidium iodide staining

Alexa Fluor 488 labeled-Annexin-V and propidium iodide (PI) staining were utilized as biomarkers for the detection of apoptosis and necrosis in ADPKD and NHK cells by flow cytometric analysis, following the manufacturer's protocols (Invitrogen, Carlsbad, CA, USA). Briefly, cells were trypsinized, washed in PBS, and resuspended in binding buffer (50 mM HEPES, 700 mM NaCl, 12.5 mM CaCl_2_, pH 7.4). After addition of 5 μL Annexin-V and 1 μL PI, cells were incubated at 37°C and protected from light for 15 min. Samples were diluted with binding buffer and were analyzed using a LSRII flow cytometer (Beckton Dickinson, Franklin Lakes, NJ). Alexa Fluor 488 labeled-Annexin-V, which detects changes in distribution of phosphatidylserine and phosphatidylethanolamine at the cell plasma membrane; and propidium iodide (PI) staining, which reveals loss of cell plasma membrane integrity, allowed distinguishing cells undergoing early and late stages of apoptosis, and necrosis (Ravichandran, 2010).

### Measurement of fragmented DNA by TUNEL assay

Cells cultured on glass coverslips were treated with 3 nM ouabain for 24 h and analyzed for apoptosis using the DeadEnd fluorometric TUNEL system (Promega, Madison, WI). This method determines nuclear fragmentation, an important biochemical marker for cell apoptosis, by using terminal deoxynucleotidyl transferase to transfer fluorescein (FITC)-12-dUTP to the free 3′-OH of cleaved DNA. Cells were counter-stained with DAPI to label the nuclei. The percentage of cells undergoing apoptosis was determined using fluorescence microscopy. Carbobenzoxy-valyl-alanyl-aspartyl-[O-methyl]-fluoro-methylketone (z-VAD-fmk), at a concentration of 20 μM and camptothecin, at 5 μM (Promega Corporation, Madison, WI), were applied as an inhibitor and inducer of apoptosis, respectively.

### Immunoblot analysis

Cells treated with and without 3 nM ouabain for 24 h were washed once with ice-cold phosphate buffered saline (PBS) and lysed in a solution containing 10 mM Tris-Cl (pH 7.4), 10 mM NaCl, 3 mM MgCl_2_, and 0.1% NP-40. Samples were centrifuged at 10,000 × *g* for 10 min. Protein amounts were determined by Bio-Rad Protein Assay (Bio-Rad, Hercules, CA). Fifty μg of the cleared lysates were subjected to SDS-PAGE (15% gel) and blotted on to nitrocellulose membranes. Immunoblots were probed with different primary antibodies that recognize PARP-1, BCL-2, BAX, caspase-3 or caspase-8 (Cell Signaling Technology, Boston, MA). Species-specific secondary antibodies conjugated to horse-radish peroxidase and enhanced chemiluminescence was used for protein detection (Santa Cruz Biotechnology, Dallas, TX). Protein expression levels were determined by densitometry and were expressed as a ratio of the corresponding untreated controls.

### Cytochrome c analysis

Cytochrome c release from mitochondria was studied in NHK and ADPKD cells after treatment with or without 3 nM ouabain for 24 h by immunoblot and immunocytochemistry. For the immunoblot analysis, cells were harvested and cytosolic or mitochondrial fractions were prepared using the Cell Fractionation Kit ab109719, according to the manufacturer's instructions (AbCam, Cambridge, UK). Samples were subjected to SDS/PAGE and proteins transferred to nitrocellulose membranes. Cytochrome c was determined using a monoclonal antibody from BD Biosciences (San Diego, CA) and horse-radish peroxidase conjugated secondary antibodies; and its levels were estimated by densitometric analysis of the bands obtained from the cytoplasmic fractions. For the immunocytochemical analysis, cells were plated on coverslips and treated with or without ouabain for 23.5 h. Then, 100 nM MitoTracker Red CMXRos (Thermo Fisher Scientific, Waltham, MA) was added to the cells and they were incubated for an additional 30 min at 37°C, protected from light. Cells were fixed with 3.7% paraformaldehyde in serum-free media for 15 min at 37°C. Samples were washed in PBS and permeabilized with acetone for 5 min. Anti-cytochrome c antibody (BD Biosciences, San Diego, CA) (1:75) was applied to the cells overnight at 4°C. Coverslips were washed once with PBS, and then slides were incubated with secondary Alexa 488-conjugated antibodies for 1 h at room temperature. Samples were washed in PBS and mounted onto microscope slides with DAPI Slowfade Gold solution (Thermo Fisher Scientific, Grand Island, NY). Slides were viewed using a Eclipse 80i Upright microscope (Nikon Instruments, Inc., Melville, NY). Analysis of cytochrome c release from the mitochondria was quantified as the ratio of cytochrome c release was determined from the obtained images, by quantifying the number of pixels in the cell cytosol divided by the number of pixels in mitochondria. Values were expressed as the ratio of cytosolic/mitochondrial cytochrome c levels, as previously described (Gao et al., 2001). This allowed characterizing the mitochondrial to cytosolic distribution of cytochrome c.

### Caspase-3/7 activity determination

Caspase-3/7 activity was determined using the Caspase-Glo 3/7 Assay according to manufacturer's instructions (Promega, Madison, WI). Briefly, NHK and ADPKD cells were plated into black-walled, clear-bottomed 96-well plates (Corning Inc., Corning, NY) at a density of 4000 cells per well and treated with or without ouabain for 24 h. The luminescent caspase-3/7 substrate was added following the manufacturers specifications. The luminescent signal resulting from cleavage of the substrate specific to caspase-3 or -7 is proportional to the amount of caspase activity present. Data were expressed as a percentage of untreated controls.

### Data analysis

Statistical significance of the differences between ouabain treated and untreated controls was determined by Student's *T*-test. Also, Bonferroni's test was applied as another way to confirm statistically significant differences. Statistical significance was defined as *P* < 0.05.

## Results

### Ouabain stimulates apoptosis in ADPKD but not in NHK cells

**Figure 1:**
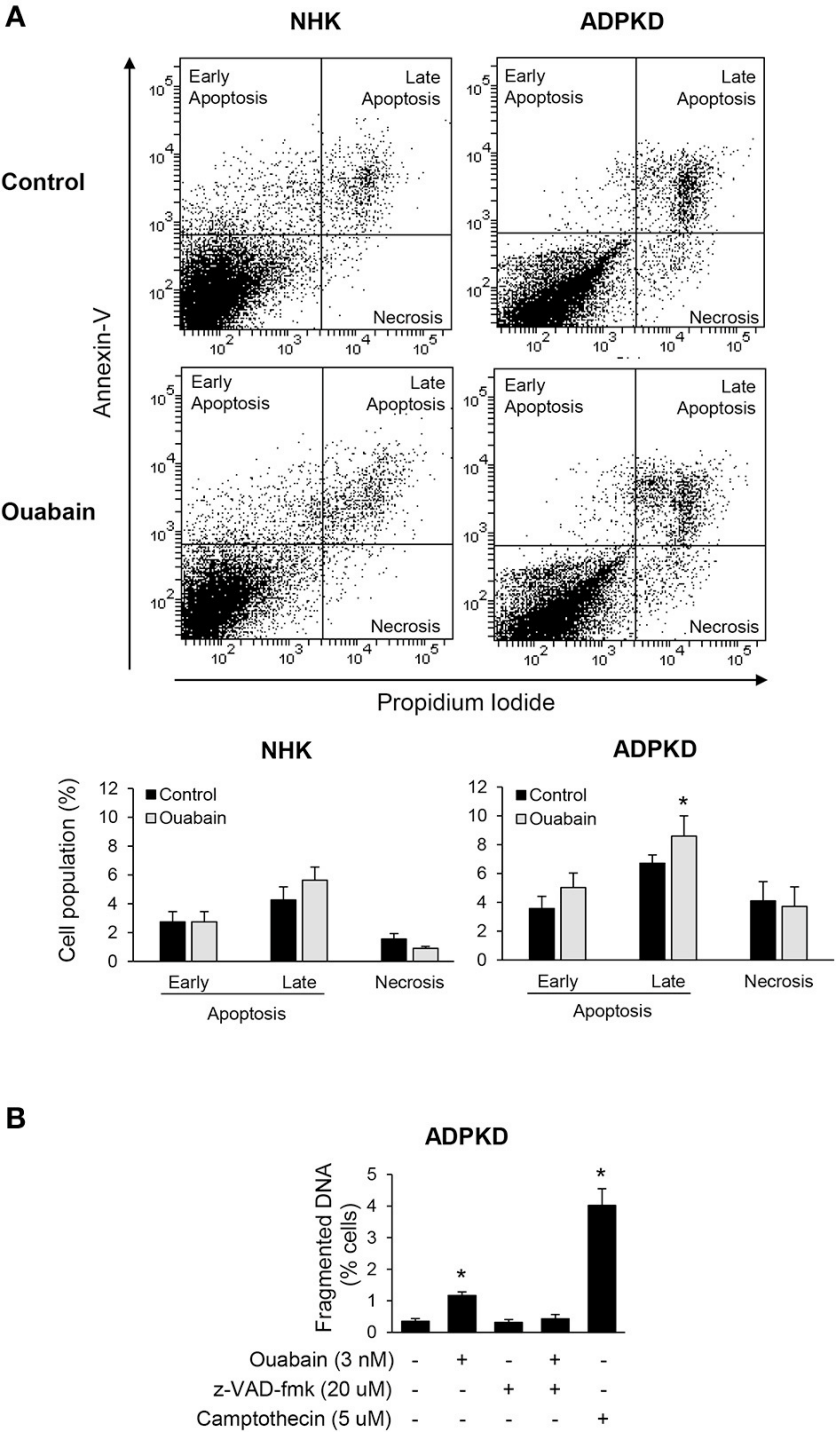
**Ouabain induces apoptosis in ADPKD, but not in NHK cells. (A)** Alexa Fluor 488 labeled-Annexin V and PI staining. After treatment with ouabain for 24 h, cells were labeled and sorted using flow cytometry. The top panel shows representative plots for the sorted NHK and ADPKD cells, without and with of ouabain. Different cell populations undergoing necrosis, early and late apoptosis, identified by differential annexin-V/PI labeling, were quantified and expressed as percent of total cells (bottom panel). **(B)** DNA fragmentation assays. Cells seeded onto glass coverslips were incubated with 3 nM ouabain and the indicated experimental conditions for 24 h, fixed and analyzed for apoptosis using the Dead-End Fluormetric TUNEL System. Fragmented DNA was labeled by incorporation of fluorescein-12-dUTP(a) at 3′-OH DNA ends. The nuclei were counterstained with DAPI and visualized by immunofluorescence microscopy. At least 10 random fields were analyzed from each of three different ADPKD kidneys. In all graphs, bars represent the mean ± SEM of three different experiments. The asterisks indicate values that are statistically different compared to the corresponding untreated control, with *P* < 0.05.

Ouabain has been reported to stimulate apoptosis in a cell type specific manner (Silva and Soares-da-Silva, 2012). We explored weather ouabain affected programmed cell death in NHK and ADPKD cells. For this, we treated NHK and ADPKD cells with 3 nM ouabain, a concentration of this hormone that is within the levels commonly found to be circulating in blood. Twenty-four hours later, we determined cell apoptosis and necrosis using Alexa Fluor 488 labeled-Annexin-V and PI labeling and flow cytometry. Sorting of the cells based on these markers showed that ouabain treatment caused a modest but significant increase in the number of ADPKD cells undergoing apoptosis compared to untreated controls (Figure 1A, top panel). This increase corresponded to cells showing signs of late apoptosis, while the number of ADPKD cells showing early manifestations of apoptosis, or undergoing necrosis did not significantly change with ouabain administration (Figure 1A, bottom panel). Different from ADPKD cells, ouabain did not induce programmed cell death (either early or late apoptosis), or necrosis in NHK cells (Figure 1A, top and bottom panels).

To further estimate apoptosis, we determined DNA nuclear fragmentation by TUNEL assay in NHK and ADPKD cells. In agreement with the Alexa Fluor 488 labeled-Annexin-V and PI studies, ouabain slightly, but statistically significantly increased TUNEL staining in ADPKD cells (Figure 1B). This ouabain-induced increase in DNA fragmentation could be rescued by the pan-caspase inhibitor, carbobenzoxy-valyl-alanyl-aspartyl-[O-methyl]-fluoro-methylketone (z-VAD-fmk), further suggesting that ouabain is inducing apoptosis in ADPKD cells. The topoisomerase I inhibitor, camptothecin, was used as a positive control for apoptosis (Figure 1B). Altogether, these experiments show that ouabain stimulates apoptosis in ADPKD, but not in NHK cells.

### Ouabain modulates expression of BCL-2 protein family members in ADPKD cells

**Figure 2:**
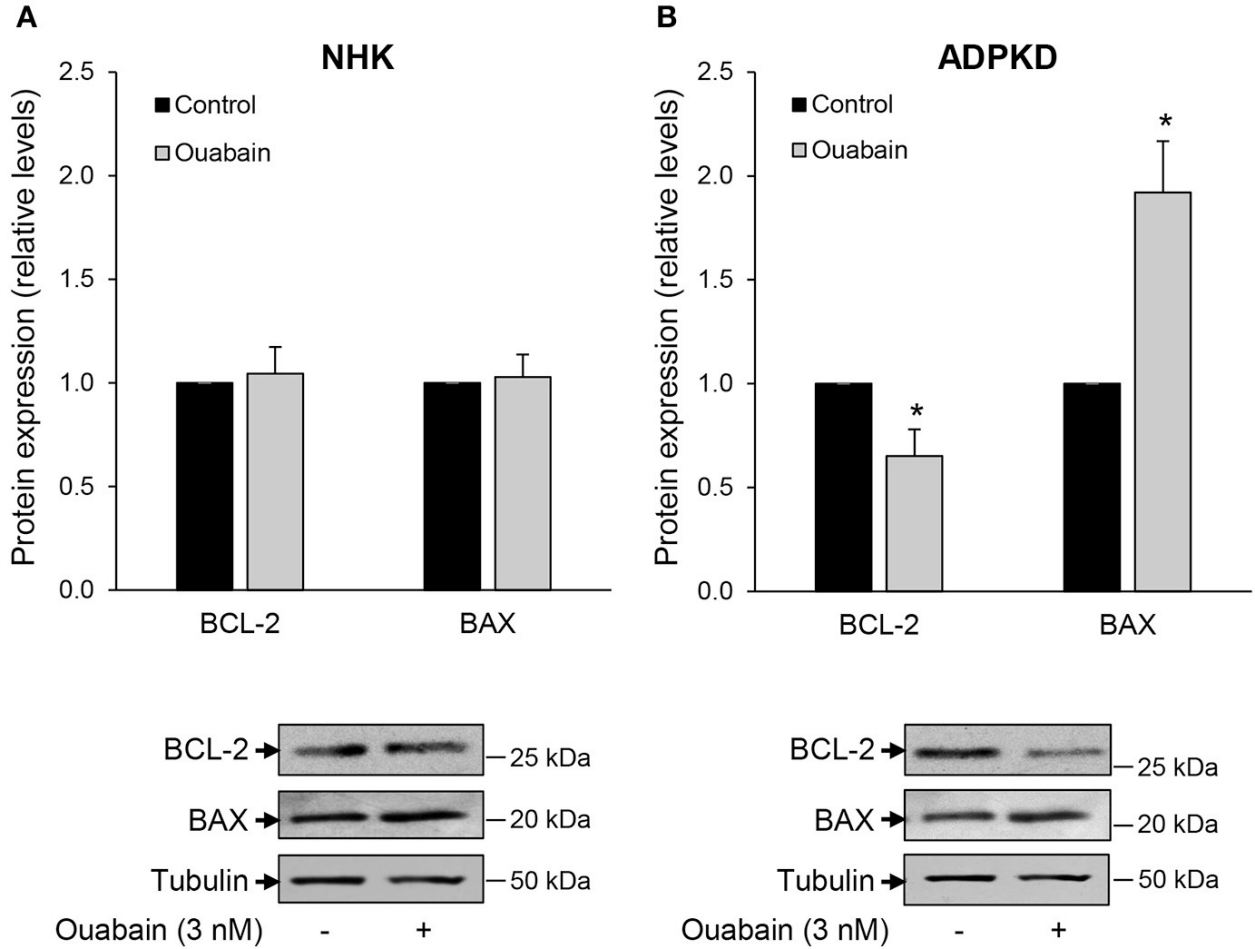
**Ouabain modifies the expression of BCL-2 and BAX protein levels in ADPKD, but not NHK cells**. NHK **(A)** and ADPKD **(B)** cells were treated in the absence and presence of 3 nM ouabain and 24 h later, expression levels of BCL-2 and BAX were determined by immunoblot. Tubulin was used as a loading control. Top panels show the densitometric analysis of the protein bands, while bottom panels show representative blots. Bars are the mean ± SEM of three experiments. Asterisks indicate statistically different values, with *P* < 0.05 vs. untreated control.

Whether a cell undergoes apoptosis is in part determined by the ratio of pro- to anti-apoptotic members of the BCL-2 protein family (Green and Llambi, 2015). Within the BCL-2 members, BAX and BAK function as pro-apoptotic agents, while BCL-2 behaves as an anti-apoptotic mediator (Zheng et al., 2015). To further characterize the mechanisms by which ouabain induces cell death in renal normal and cystic cells, we examined the role of these apoptotic regulators in NHK and ADPKD cells. Ouabain treatment for 24 h did not alter the expression levels of either BAX or BCL-2 proteins in NHK cells (Figure 2A). However, in ADPKD cells, ouabain caused a significant decrease in the anti-apoptotic BCL-2 protein, with a concomitant increase in the pro-apoptotic BAX protein levels (Figure 2B). This change toward a pro-apoptotic protein ratio supports the role of ouabain as an inducer of apoptosis in ADPKD cells, and suggests that its effects are mediated via the intrinsic pathway of programmed cell death.

### Ouabain enhances cytochrome C release from ADPKD cell mitochondria

**Figure 3:**
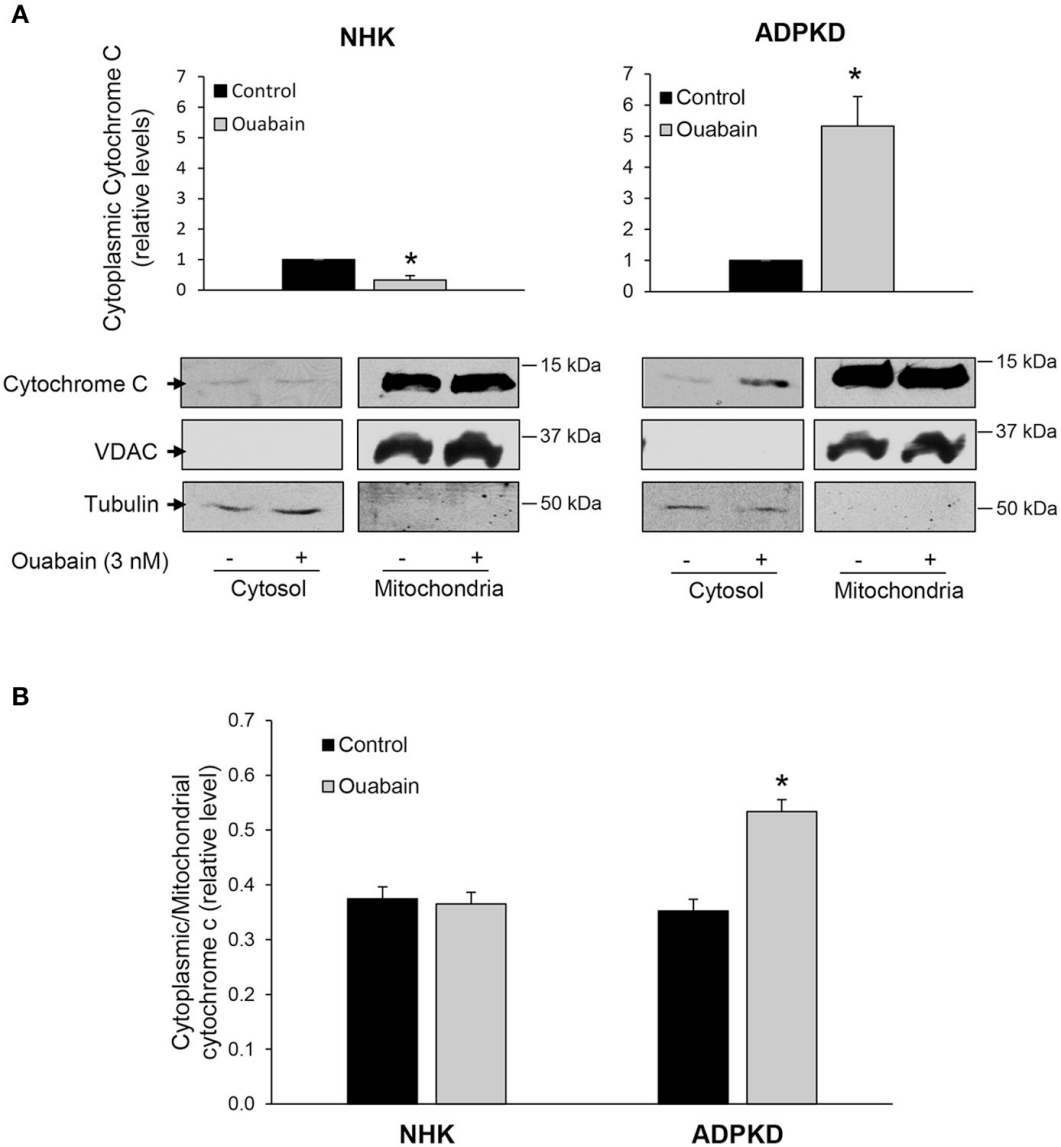
**Ouabain stimulates cytochrome C release from mitochondria in ADPKD, but not NHK cells. (A)** Immunoblot analysis. After treatment with or without 3 nM ouabain for 24 h, cells were processed to obtain cell cytoplasmic and mitochondrial fractions, and samples were subjected to immunoblot analysis to determine cytochrome c levels. Tubulin was used as a loading control, while VDAC was used as a mitochondrial marker. The bottom panels show representative immunoblots. Relative densitometric levels for cytochrome c in the cytoplasm are shown in the upper panels and they represent data compiled from four different experiments. Due to the difference between mitochondrial and cytoplasmic cytochrome c levels, and to better quantify the cytoplasmic bands, different exposure times for the cytochrome c cytosolic and mitochondrial samples from the same immunoblots were used. **(B)** Immunocytochemical analysis. After treatment with 3 nM ouabain for 24 h, NHK and ADPKD cells were labeled for cytochrome c and MitoTracker, to visualize mitochondria. Cytochrome c release was quantified and expressed as the cytosol to mitochondrial ratio. Bars represent the compiled data from 3 different experiments. In A and B, bars represent the mean ± SEM. Asterisks indicate statistically different values, with *P* < 0.05 vs. untreated control.

A pro-apoptotic change in BCL-2/BAX protein ratio is commonly followed by the release of cytochrome c from mitochondria, another event in the activation cascade of programmed cell death (Li and Dewson, 2015; Um, 2015). Therefore, we determined the release of cytochrome c from mitochondria, by measuring the levels of cytochrome c in cytoplasmic fractions from NHK and ADPKD cells by immunoblot. As expected, and as shown in Figure 3A, cytochrome c levels were much higher in the mitochondrial than in the cytosolic fractions of both NHK and ADPKD cells. Importantly, cytochrome c was significantly augmented by ouabain in cytosolic fractions from ADPKD, but it was slightly decreased in NHK cells (Figure 3A). Due to the disparity between the high saturating levels of cytochrome c, typical of mitochondria, and the normal low levels of cytochrome c found in cytoplasm, we used higher exposure times for the development of the immunoblots corresponding to the cytosolic than the mitochondrial cell fractions from the same blot. This allowed us to perform a better quantification of the cytochrome c bands in the cell cytosol; which, along with the sole presence of VDAC in mitochondria, support the lack of any major cross contamination between mitochondrial and cytoplasmic samples. The increase in cytochrome c in the cytoplasm suggests that the change in BCL-2/BAX ratio in ADPKD cells did result in release of cytochrome c from the cell mitochondria.

In addition, changes in cytochrome c localization were studied by immunocytochemistry. For this, cells were labeled with MitoTracker Red, a dye which allows the visualization of mitochondria, and an anti-cytochrome c. Then, the ratio of cytoplasmic to mitochondrial localization of cytochrome c was determined by quantification of pixel density as described (Gao et al., 2001). As shown in Figure 3B, the ratio of cytoplasmic/mitochondrial cytochrome c did not change with ouabain treatment in NHK cells. In contrast, this ratio was significantly increased by ouabain in ADPKD cells (Figure 3B). Altogether, these results show that ouabain promotes the release of cytochrome c from the mitochondria of ADPKD cells and agree with the notion that ouabain induces apoptosis in these cells.

### Ouabain activates caspase-3/7 in ADPKD cells

**Figure 4:**
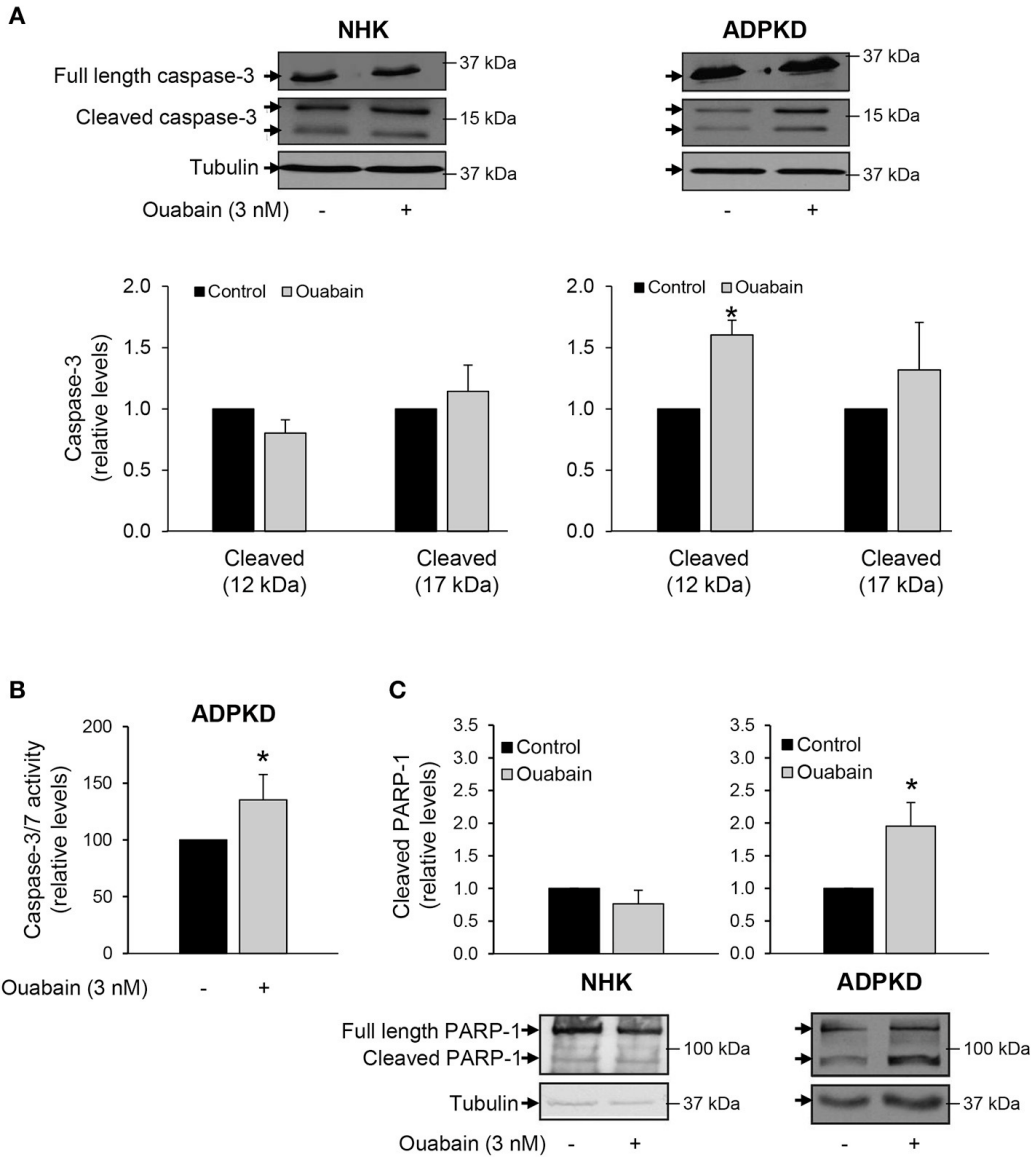
**Ouabain stimulates caspase-3 cleavage and activity in ADPKD, but not NHK cells. (A)** Caspase-3 cleavage. After treatment with 3 nM ouabain for 24 h, the total (35 kDa) and cleaved products (17 and 12 kDa) of caspase-3 were determined in NHK and ADPKD cells by immunoblot and densitometric analysis. Upper panels show representative blots and bottom panels the densitometric analysis from three different experiments. Bars are the mean ± SEM of three experiments. **(B)** Relative caspase-3 and -7 activity levels. ADPKD cells were treated in the absence and presence of 3 nM ouabain and caspase activity was measured using the Caspase-3/7 Glo Assay. Data are the mean ± SEM of sextuplicate experiments. **(C)** PARP-1 cleavage. After ouabain treatment for 24 h, NHK and ADPKD cell lysates were subjected to immunoblot to determine fragmentation of the caspase-3 substrate, PARP-1. The top panels show the densitometric analysis of the total and cleaved (89 kDa) PARP-1 bands. Cleaved PARP-1 is expressed relative to the corresponding untreated controls. The bottom panels show representative immunoblots. Bars represent the mean ± SEM of 5 different experiments. Asterisks show statistically different values, compared to untreated controls and with *P* < 0.05.

Proteases of the caspase family play an essential role in the cleavage of specific substrates that mediate cell apoptosis (Zhivotovsky, 2003; Poreba et al., 2013). To assess the involvement of caspases in ouabain-induced apoptosis of ADPKD cells, we treated NHK and ADPKD cells in the presence and absence of 3 nM ouabain for 24 h and determined activation of the “executioner” caspase-3 via its cleavage status. Caspase-3 cleavage was determined by immunoblot. As shown in Figure 4A, caspase-3 cleavage was not modified by ouabain in NHK cells (left panels). In contrast, in ADPKD cells, ouabain significantly increased the cleavage of pro-caspase-3 into its active large (p17) and small (p12) fragments (right panels).

In addition, we directly measured the levels of caspase-3 activity, in ADPKD cells treated with and without 3 nM ouabain. Once activated, caspase-3 cleaves the same peptide sequences than caspase-7 and their activities cannot be distinguished with the assay that we used. In any case, both caspase-3 and -7 are “executioner” caspases, involved in downstream cleavage of substrates that mediate many of the typical biochemical and morphological events of apoptosis (Zhivotovsky, 2003; Poreba et al., 2013). As shown in Figure 4B, ouabain significantly stimulated caspase-3/7 activity of ADPKD cells.

Ouabain dependent caspase-3 activation by ouabain was also estimated by immunoblot analysis of poly (ADP-ribose) polymerase-1 (PARP-1), a known target of caspase-3 action (Kauppinen and Swanson, 2007). Consistent with activation of caspase-3, ouabain increased the cleavage of PARP-1 in ADPKD cells, but not in NHK cells (Figure 4C).

### Ouabain does not activate the extrinsic pathway of ADPKD cell apoptosis

**Figure 5:**
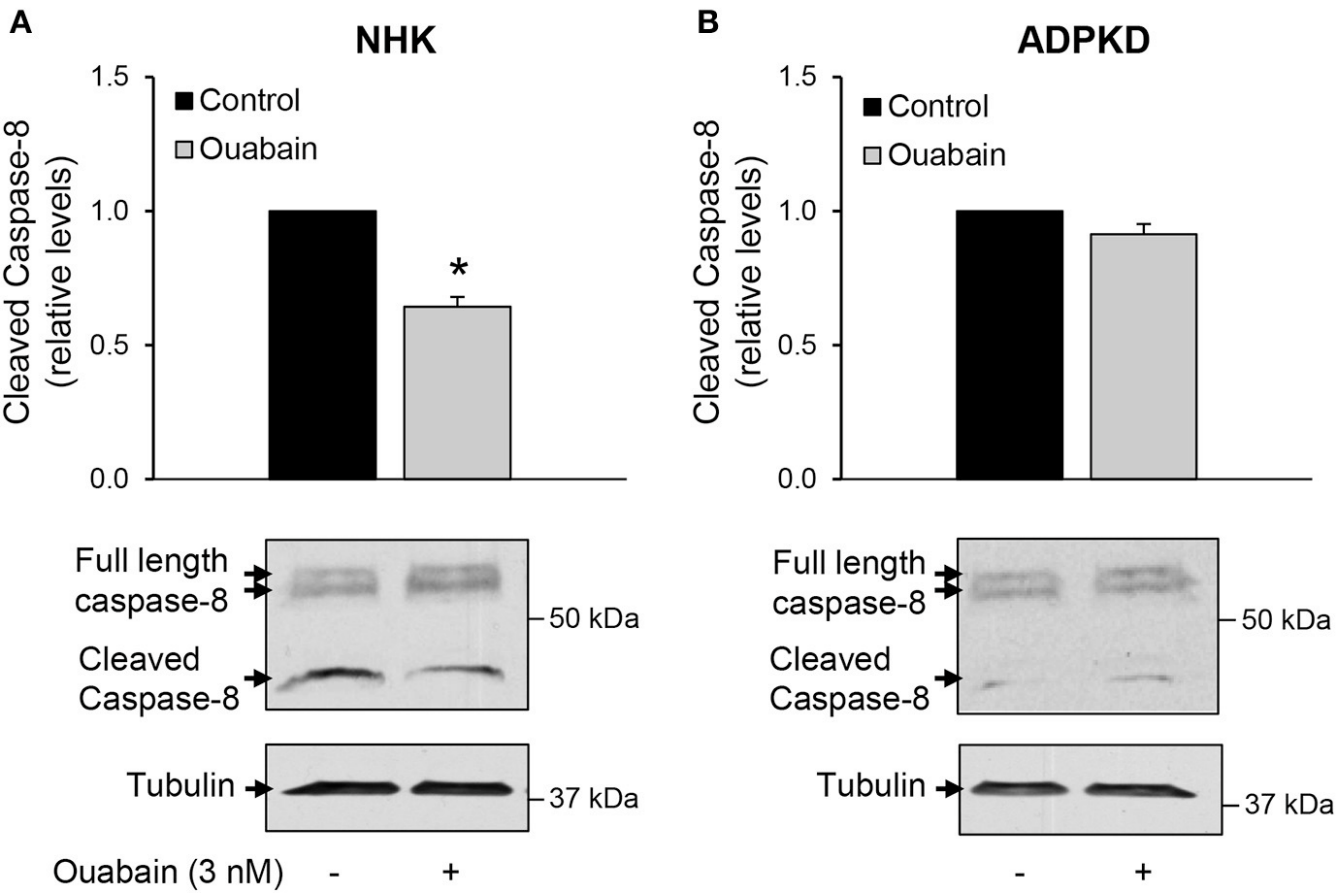
**Ouabain does not affect the extrinsic pathway for apoptosis in ADPKD cells**. NHK **(A)** and ADPKD **(B)** cells were treated with 3 nM ouabain for 24 h and the total (41/43 kDa) and cleaved (37 kDa) forms of caspase-8 were determined by immunoblot analysis and quantified by densitometry. The top panels show the densitometric analysis of the bands from 4 different experiments. The bottom panels show representative blots. Values are the mean ± SEM. Asterisks indicate statistically different values, with *P* < 0.05 vs. untreated control.

The factors involved in triggering cell apoptosis can act through two main mechanisms, the intrinsic and extrinsic pathways (Green and Llambi, 2015). Our findings that ouabain stimulates the release of cytochrome c from mitochondria suggest a role for the intrinsic pathway in the mechanisms leading to ouabain-mediated apoptosis in ADPKD cells. To determine the involvement of the extrinsic pathway in ouabain-induced ADPKD apoptosis, we measured the activation of caspase-8. This protease is involved in the extrinsic pathway of programmed cell death and its cleavage is a marker for its activation (Salvesen, 1999). We treated NHK and ADPKD cells in the presence and absence of 3 nM ouabain for 24 h and determined the total and cleaved forms of caspase-8 by immunoblot. Compared to untreated controls, ouabain unexpectedly, decreased caspase-8 cleavage in NHK cells (Figure 5A), but it did not affect caspase-8 cleavage in ADPKD cells (Figure 5B). These results suggest that the apoptotic effects of ouabain in ADPKD cells are not mediated by caspase-8 and the extrinsic pathway of programmed cell death. In addition, the reduction in caspase-8 in NHK cells suggests a protective effect of ouabain toward apoptosis in normal cells.

### The effect of ouabain on ADPKD apoptosis is independent from cell proliferation

**Figure 6:**
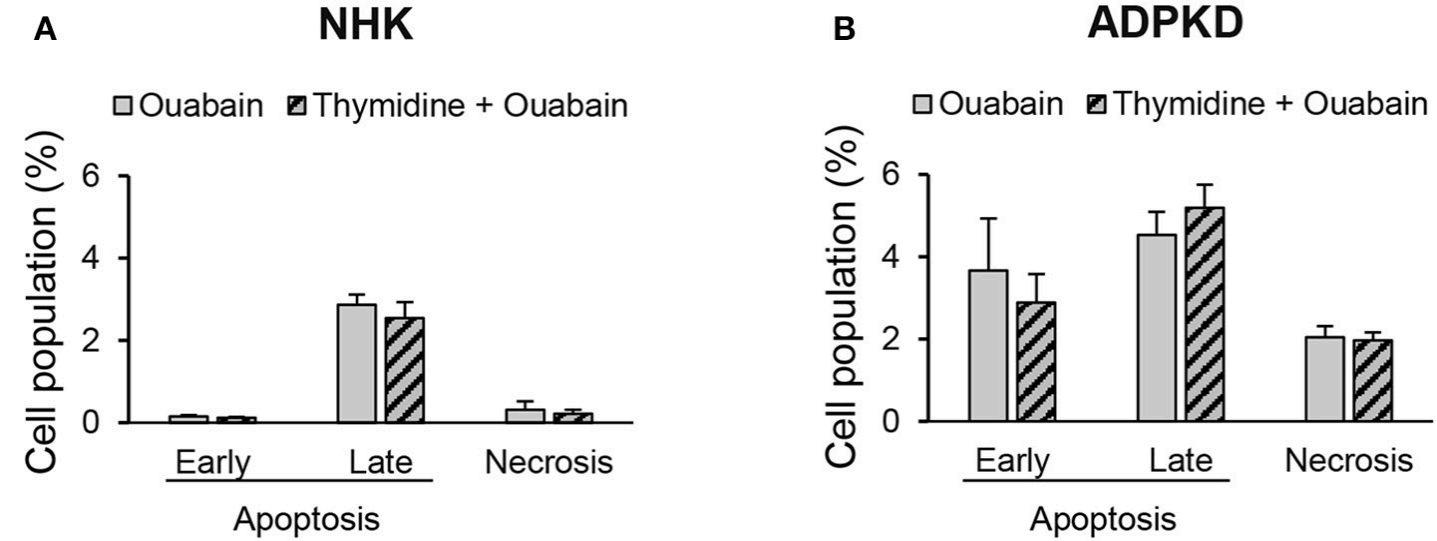
**Ouabain induces ADPKD cell apoptosis independent from its proliferative effects**. NHK **(A)** and ADPKD **(B)** cells were treated with or without 3 nM ouabain, in the presence and absence of 2.5 M thymidine to arrest cell growth. Alexa fluor-labeled-annexin-V and PI staining and cell sorting were utilized to detect apoptosis/necrosis. Bars represent the mean ± SEM of 3 different experiments. While the values for ouabain induced apoptosis were statistically significant between NHK and ADPKD cells (*P* < 0.05), no statistical differences were found between cells treated with ouabain in the presence and absence of thymidine, both for NHK or ADPKD samples.

We have previously shown that ouabain enhances the growth of ADPKD cells (Nguyen et al., 2007). The ouabain-induced activation of apoptosis that we observe in ADPKD cells could just be secondary to the high proliferative effects that ouabain has in these cells. To investigate this possibility, we inhibited cell proliferation in NHK and ADPKD cells with thymidine, and treated the cells with 3 nM ouabain for 24 h. Finally, we determined apoptosis levels by cell sorting, after labeling the cells with Alexa Fluor 488 labeled-Annexin-V. As shown in Figure 6A, ouabain had no effect on NHK cell early or late apoptosis, either in the presence of absence of thymidine. In ADPKD cells, ouabain activated programmed cell death, even when cell proliferation was blocked with thymidine (Figure 6B). This shows that the effects of ouabain on ADPKD cell apoptosis directly target programmed cell death and they are not an indirect consequence of the exacerbated growth that the hormone causes in the cells.

## Discussion

In this work we have shown that ouabain stimulates apoptosis in human ADPKD cells. Thus, ouabain causes changes in plasma membrane phospholipids, induces DNA fragmentation, alters the balance of BCL-2 protein expression, favors release of cytochrome c from mitochondria, and activates caspase-3/7; all of which are typical events of programmed cell death. We found that the levels of apoptosis that ouabain promotes are modest; however, this agrees with the apoptosis seen in ADPKD cells, which cannot compensate for the proliferative nature of the disease (Woo, 1995; Lanoix et al., 1996). Also, we found that apoptosis levels varied depending on the method used for cell death determination, being slightly higher when Alexa Fluor 488 labeled-Annexin-V/PI was used, as compared to tunel assay. This may reflect differences in the end points measured in each case (plama membrane vs. DNA changes respectively), or differences in the sensitivity of each assay. Ouabain-induced ADPKD apoptosis occurs even when cell proliferation in the cells is blocked. This suggests that the enhancement of ADPKD cell apoptosis by ouabain is due to a direct action of ouabain on programmed cell death, and not a secondary consequence of increased cell growth, which is another effect induced by ouabain in these cells (Blanco and Wallace, 2013). The role of ouabain on apoptosis has been shown to be cell type dependent (Silva and Soares-da-Silva, 2012). Similar to ADPKD cells, various cancer cell types either undergo apoptosis when treated with ouabain, or are sensitized for apoptosis triggered by other compounds (Huang et al., 2004; Winnicka et al., 2007; Qiu et al., 2008; Bloise et al., 2009; Xu et al., 2010; Platonova et al., 2011; Alonso et al., 2013; Chen et al., 2014; Wang et al., 2014). Therefore, ADPKD are not the only diseased cells that react to ouabain with an apoptotic response.

We show that, different from ADPKD cells, ouabain does not induce apoptosis in NHK cells. In agreement with these observations, ouabain does not trigger programmed cell death in renal proximal tubule cells and explanted embryonic kidneys from normal rats. Moreover, in those studies, ouabain was shown to have a protective effect against apoptosis induced by serum-starvation (Li et al., 2010) and Shiga toxin-2 infection (Burlaka et al., 2013). While the goal of our experiments was not to explore the effects of ouabain under stimuli that challenge the cells to undergo apoptosis, we find a decrease in cytochrome c release from mitochondria, a reduction of ouabain-induced caspase-8 cleavage, and a trend for lower caspase-3 cleavage in NHK cells after ouabain treatment. This suggests that ouabain may be shifting NHK cells toward a pro-survival phenotype. In any case, it appears that, despite the differences in species and renal origin, ouabain acts as a pro-survival agent in normal kidney cells.

Interestingly, increased apoptosis is a feature of early and late stages of human ADPKD (Woo, 1995; Lanoix et al., 1996), and it is linked to cystogenesis in various animal models of polycystic kidney disease (Moser et al., 1997; Trudel et al., 1998; Lin et al., 1999; Lager et al., 2001). ADPKD apoptosis has been shown to take place in the epithelial cells lining the kidney cysts, and in cysts formed by Madin-Darby canine kidney cells (MDCK) grown in collagen matrix, a model of kidney cystic disease (Woo, 1995; Lin et al., 1999; Ecder et al., 2002; Ibrahim, 2007). Moreover, polycystin 1, the primary gene altered in ADPKD, has been shown to be a regulator of programmed cell death in renal cells, and its over-expression confers the host cells resistance to apoptosis (Boletta et al., 2000). Apoptosis has also been detected in non-cystic tissue in ADPKD kidneys, which suggests that programmed cell death of the normal remaining kidney contributes to the progressive deterioration of ADPKD renal function (Woo, 1995; Ibrahim, 2007; Goilav et al., 2008). Therefore, it appears that apoptosis is an essential concomitant event that helps kidney cyst development, and along with cell proliferation contributes to the pathophysiology of ADPKD. Our results show that in human cells, the target for ouabain-induced apoptosis is the cystic, ADPKD cells, and not the NHK cells. The apoptotic effect of ouabain in ADPKD cells, along with the aberrant increase that ouabain promotes in ADPKD cell growth (Nguyen et al., 2007), places ouabain as a modulator of two essential mechanisms involved in the pathophysiology of ADPKD. It is clear that that the increase in apoptosis caused by ouabain is not sufficient to overcome the ouabain-induced ADPKD cell growth. Therefore, ouabain functions as a factor that creates a dysregulation of ADPKD cell growth/death, in favor of cell proliferation.

Our studies used amounts of ouabain that are within those circulating in plasma (Bagrov et al., 2009). This highlights the relevance of ouabain as a factor that contributes to the pathophysiology of ADPKD. At present, the molecular basis for the different responses of ADPKD and NHK cell to ouabain is unclear; however, we have previously found that, different from NHK cells, ADPKD cells contain a fraction of the total Na,K-ATPase with an abnormal higher affinity for ouabain (Nguyen et al., 2007). Due to their increased ouabain affinity, ADPKD cells may be just more susceptible to the endogenous circulating levels of ouabain, to which NHK cells do respond to the same extent. In addition to their differential sensitivity to ouabain, ADPKD cells also differ from NHK cells in the activity of several intracellular signaling pathways. For example, it is known that the kinase B-Raf and the ERK pathway have an abnormal reactivity to different circulating factors in ADPKD cells (Yamaguchi et al., 2003; Rajagopal and Wallace, 2015). It is therefore possible that, in ADPKD cells, ouabain impinges on pathways which respond in an exacerbated manner on apoptotic effectors to cause programmed cell death. Undoubtedly, more studies are necessary before we can fully understand the molecular mechanisms by which ADPKD cells respond differently to the variety of stimuli that circulate in blood, including ouabain.

As shown from our labeling experiments with PI, ouabain does not cause cell necrosis, which suggests that ouabain effects are non-toxic for the ADPKD cells. This agrees with the notion that, in ADPKD cells, ouabain is not completely inhibiting Na,K-ATPase ion transport, but that at relatively low, physiologic amounts, it activates downstream effectors, as we have shown before (Nguyen et al., 2011). In further support of this, we here found that ouabain activates several mediators in the signaling pathway that leads to apoptosis. Thus, in ADPKD, but not NHK cells, ouabain causes an inbalance in the expression of the BCL-2 and BAX proteins, involved in anti- and pro-apoptotic effects respectively. Ouabain slightly inhibited BCL-2 expression and augmented BAX levels, agreeing with induction of apoptosis in the cells. Interestingly, BCL-2 deficient mice show increased kidney apoptosis and the development of renal cyst disease (Veis et al., 1993). The downregulation of BCL-2 and the pro-apoptotic effects of ouabain may represent mechanisms, which together with those on cell growth and fluid secretion, contribute to the enhancement of the cystic phenotype of ADPKD cells. The decrease in BCL-2 protein concentration in response to ouabain is not unique to ADPKD cells and it has been found in other cell types (Kulikov et al., 2007; Sapia et al., 2010; Trenti et al., 2014). Moreover, overexpression of either BCL-2 or BCL-XL has been reported to abrogate the pro-apoptotic effects of ouabain in a lymphoma cell line (Gilbert and Knox, 1997). Interestingly, the involvement of the BCL-2 family of proteins in ouabain-mediated effects in cells is also supported by the finding that the Na,K-ATPase α subunit contains BH1- and BH3-like motifs, similar to those involved in the pairing of BCL-2 family proteins among each other (Zha et al., 1996; Lauf et al., 2015), and that the BCL-2 family member proteins, BCL-XL and BAK1 co-immunoprecipitate with Na,K-ATPase in A549 lung cancer cells and in fetal human epithelial lens cells (Lauf et al., 2015). While it is unknown whether Na,K-ATPase acts as a scaffolding protein to mediate pro-apoptotic effects via its BH1- and BH3-motifs in ADPKD cells, it is clear that BCL family proteins are involved in ouabain-induced apoptotic effects in the renal epithelial cystic cells.

Besides stimulating the expression of BCL-2 protein, ouabain also impacts on other important mediator of apoptosis in ADPKD cells, such as the mitochondria, through the release of cytochrome c. Moreover, ouabain activates the executioner caspases 3 and 7. Instead, ouabain does not activate the cleavage of caspase-8, an essential effector of the extrinsic apoptotic pathway. Therefore, ouabain induces ADPKD cell apoptosis via specific activation of the intrinsic pathway of programmed cell death. Ouabain has been shown to activate the intrinsic apoptotic pathway in other cell types (Xiao et al., 2002; Kulikov et al., 2007; Alonso et al., 2013). Importantly, our results concur with studies in the Han:SPRD rat, a rodent model of ADPKD, in which the increase in apoptosis is dependent on activation of the intrinsic pathway of programmed cell death (Edelstein, 2005; Tao et al., 2005). In this manner, ouabain enhances an apoptotic route, which commonly participates in ADPKD programmed cell death.

In summary, we have further advanced our understanding of the effects of ouabain in ADPKD cells and found that physiological amounts of ouabain stimulate apoptosis in these cells through activation of the intrinsic pathway of programmed cell death. Activation of this characteristic event of ADPKD, together with other enhancing actions of ouabain on the ADPKD phenotype (Blanco and Wallace, 2013), further supports the role of ouabain as a non-genetic factor that can modulate renal cystogenesis and the progression of ADPKD.

## Author contributions

Conception and design: GB; completion of experiments: JV; analysis and interpretation of data: JV and GB; drafting the article: JV; article revision and approval of the final version of the manuscript: JV and GB.

### Conflict of interest statement

The authors declare that the research was conducted in the absence of any commercial or financial relationships that could be construed as a potential conflict of interest.
